# Neuronal Spike Timing Adaptation Described with a Fractional Leaky Integrate-and-Fire Model

**DOI:** 10.1371/journal.pcbi.1003526

**Published:** 2014-03-27

**Authors:** Wondimu Teka, Toma M. Marinov, Fidel Santamaria

**Affiliations:** UTSA Neurosciences Institute, The University of Texas at San Antonio, San Antonio, Texas, United States of America; École Normale Supérieure, College de France, CNRS, France

## Abstract

The voltage trace of neuronal activities can follow multiple timescale dynamics that arise from correlated membrane conductances. Such processes can result in power-law behavior in which the membrane voltage cannot be characterized with a single time constant. The emergent effect of these membrane correlations is a non-Markovian process that can be modeled with a fractional derivative. A fractional derivative is a non-local process in which the value of the variable is determined by integrating a temporal weighted voltage trace, also called the memory trace. Here we developed and analyzed a fractional leaky integrate-and-fire model in which the exponent of the fractional derivative can vary from 0 to 1, with 1 representing the normal derivative. As the exponent of the fractional derivative decreases, the weights of the voltage trace increase. Thus, the value of the voltage is increasingly correlated with the trajectory of the voltage in the past. By varying only the fractional exponent, our model can reproduce upward and downward spike adaptations found experimentally in neocortical pyramidal cells and tectal neurons in vitro. The model also produces spikes with longer first-spike latency and high inter-spike variability with power-law distribution. We further analyze spike adaptation and the responses to noisy and oscillatory input. The fractional model generates reliable spike patterns in response to noisy input. Overall, the spiking activity of the fractional leaky integrate-and-fire model deviates from the spiking activity of the Markovian model and reflects the temporal accumulated intrinsic membrane dynamics that affect the response of the neuron to external stimulation.

## Author Summary

Spike adaptation is a property of most neurons. When spike time adaptation occurs over multiple time scales, the dynamics can be described by a power-law. We study the computational properties of a leaky integrate-and-fire model with power-law adaptation. Instead of explicitly modeling the adaptation process by the contribution of slowly changing conductances, we use a fractional temporal derivative framework. The exponent of the fractional derivative represents the degree of adaptation of the membrane voltage, where 1 is the normal leaky integrator while values less than 1 produce increasing correlations in the voltage trace. The temporal correlation is interpreted as a memory trace that depends on the value of the fractional derivative. We identify the memory trace in the fractional model as the sum of the instantaneous differentiation weighted by a function that depends on the fractional exponent, and it provides non-local information to the incoming stimulus. The spiking dynamics of the fractional leaky integrate-and-fire model show memory dependence that can result in downward or upward spike adaptation. Our model provides a framework for understanding how long-range membrane voltage correlations affect spiking dynamics and information integration in neurons.

## Introduction

The leaky integrator properties of a neuron are determined by the membrane resistance and capacitance which define a single time constant for the membrane voltage dynamics [1]–[3]. However, the voltage trace of real neurons can follow multiple timescale dynamics [4]–[6] that arise from the interaction of multiple active membrane conductances [7]–[11]. Such processes can result in power-law behavior in which the membrane voltage cannot be characterized with a single time constant [5], [12]–[15]. Since power-law dynamics can span all the scales of interest of neuronal behavior [16]–[20], it is necessary to develop a framework to study such processes and their effect on the electrical and computational capacities of neurons.

In the classical leaky integrate-and-fire model the temporal evolution of the voltage is local [21], [22]. The value of the voltage at a given time depends only on the value of the voltage in the immediate previous time step. Such a process is called Markovian. However, coupling of active conductances does not allow the value of the voltage to be memoryless [11], [17], [23]–[26]. Instead, long time correlations affect the membrane voltage for hundreds of milliseconds. The emergent effect of these membrane correlations is a non-Markovian process that can be modeled with a fractional derivative [27]–[31]. A fractional derivative represents a non-local process [32]–[34] in which the value of the variable is determined by integrating a temporal weighted voltage trace, also called the memory trace. Although fractional derivatives and integrals are almost as old as traditional calculus [32], [35], [36], they have not been widely used due to limited computer power. In the fractional integrate-and-fire model the exponent of the fractional derivative goes from 0 to 1, with 1 representing the normal derivative. As the exponent of the fractional derivative decreases, the weights of the voltage trace increase. Thus, the value of the voltage is increasingly correlated with the trajectory of the voltage in the past.

We developed and analyzed a fractional leaky integrate-and-fire model. The only parameters of the model are the conductance, capacitance, and the fractional exponent. By varying the fractional exponent our model reproduces the upward and downward spike-frequency adaptations found experimentally in pyramidal neurons [37], [38], tectal neurons [39] and fast-spiking cells of layer IV in the rat barrel cortex [40]. Furthermore, the model replicates not only the adapting firing rate but also the long first-spike latency seen in pyramidal neurons in layer V [38]. The model also produces spikes with longer first-spike latency and high inter-spike variability with power-law distribution, which cannot be reproduced by the classical integrate-and-fire model. We further analyze spike adaptation and the responses to noisy and oscillatory input. Overall, the spiking activity of the fractional integrate-and-fire model deviates from the spiking activity of the Markovian model and reflects the temporal accumulated intrinsic membrane dynamics that affect the response of the neuron to external stimulation.

## Results

The objective of this project was to develop a fractional leaky integrate-and-fire model of neuronal activity to study spiking adaptation. For this purpose we developed a fractional differential model of the leaky integrator combined with a regular spiking generation mechanism. Complex multiple timescale neuronal systems can be studied using fractional or power-law dynamics; examples range from ion channel gating properties, to diffusion of intracellular signals in Purkinje and pyramidal cells, synaptic strength and firing rate adaptation [14], [19], [27], [37], [41]–[44].

### The fractional leaky integrate-and-fire model

We define the fractional leaky integrate-and-fire model as
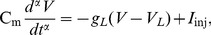
(1)along with the fire-and-reset condition
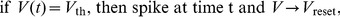
(2)where 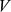 is the membrane potential, and 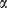 is the order (exponent) of the fractional derivative, with 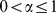. In the case of 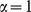, the fractional model is the same as the classical leaky integrate-and-fire model. When the membrane potential reaches a threshold (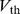), a spike is generated and 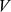 is reset to 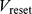 for a refractory period 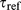. The passive membrane time constant is 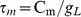. Parameter values are given in Table 1 (see Methods). For 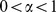, the fractional derivative of the voltage (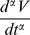 in Eq. 1) can be defined with the Caputo [45] fractional derivative
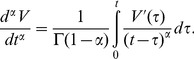
(3)


**Table 1:** Parameter values for the fractional leaky integrate-and-fire model.

Parameter	Value	Description
C_m_	0.5 nF	membrane capacitance
*V_L_*	−70 mV	leak reversal membrane potential
*V*_rest_	−70 mV	resting membrane potential
*V*_reset_	−70 mV	reset membrane potential
*V*_0_	−70 mV	initial membrane potential
*V*_th_	−50 mV	threshold membrane potential
*g_L_*	25 nS	leak conductance
*I*_inj_	3 nA	injected (applied) current
*τ*_ref_	5 ms	refractory period

For the majority of our simulations we used the parameters described in the table. However, to better fit experimental data we used in some cases 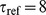 ms and 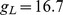 nS that results in 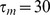 ms.

By numerically integrating the above fractional derivative (Eq. 3) using the L1 scheme [46], we approximate the fractional derivative of order 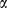, where 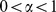,
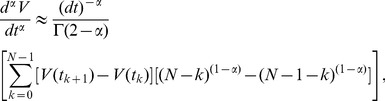
(4)where 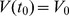, and 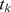 is the 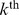 value of time such that 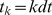. For all simulations, we use the time step 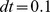 ms. By combining the right sides of Eqs. 1 and 4, and solving for 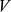 at time 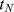 (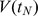) that depends on all past values of 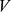 (from 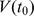 to 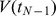), we obtain
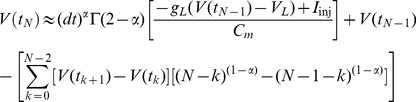
(5)where we define the Markov term weighted by the gamma function as
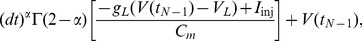
(6)and the voltage-memory trace as
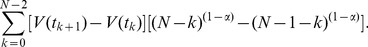
(7)


The voltage-memory trace (Eq. 7) can be further divided into the differentiation of past voltage (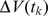) (Eq. 8) weighted by a function 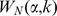 that depends on 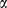 (Eq. 9):
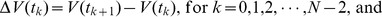
(8)

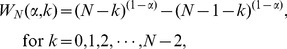
(9)where 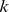 is the time counter for past events and the positive integer 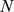 corresponds to the value of time 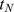 (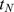 = 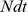) at which the voltage 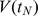 is integrated (Eq. 5).

The voltage-memory trace contains information of all the previous voltage activity of the neuron. Clearly, this is a computationally intensive problem due to the expanding matrix over time. We integrate this equation using our recently developed Fractional Integration Toolbox [47]. Since the fractional integration in Eq. 5 needs at least two inputs, 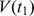 is first integrated using the classical leaky integrate-and-fire model.

### The fractional leaky integrate-and-fire model shows spike adaptation

In this section we show the spiking properties of the fractional leaky integrate-and-fire model and compare our results to experimental data, mainly from cortical pyramidal neurons.

#### Spike adaptation depends on the value of the fractional order

**Figure 1:**
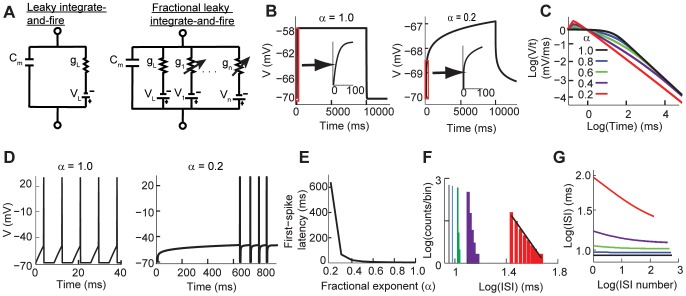
Comparison between the classical and fractional leaky integrate-and-fire models. (A) Schematic circuit diagrams for the classical (left) and fractional order (right) leaky integrate-and-fire models. (B) Sub-threshold response in the classical (left) and fractional models (right). Both stimulated with 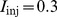 nA. (C) The sub-threshold voltage response converges to a power-law function when 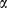 decreases. (D) While the classical model (left) generates regular spiking to a constant input, the fractional model (right) shows first spike latency and spike adaptation. Both models stimulated with 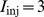 nA. (E) The first-spike latency produced by the fractional model becomes longer when 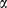 is smaller. (F) The inter-spike interval histogram as a function of 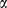. The histogram shows power-law distribution as 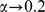. (G) The inter-spike intervals decrease over time as a function of 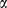. The color key in C applies to F and G.

We start by comparing the classical and fractional leaky integrate-and-fire models. The classical leaky integrator model is fully described by the cell membrane resistance and capacitance (Fig. 1A left). As mentioned before we propose that the emergent contribution of slowly varying conductances results in voltage dynamics that can be modeled with a fractional leaky integrator (Fig. 1A right). We first compared the voltage response of the model without the spiking mechanism, thus studying only the sub-threshold dynamics. In such conditions the voltage response of the fractional model to a step current using 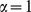 is identical to the response of the classical model (Fig. 1B left). However, as 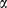 decreases the voltage response shows adaptation that is not characterized by 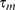 (Fig. 1B right). Instead, the voltage response increasingly shows power-law adaptation as the value of 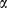 decreases (Fig. 1C). Thus, the fractional derivative transforms the sub-threshold voltage response to a constant stimulus from exponential to a power-law.

We characterized the spike adaptation of the model as a function of 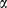 by stimulating the model with a step current and measuring the response to the first spike and the properties of the inter-spike intervals (ISIs). Our results show that the sub-threshold voltage dynamic of the fractional model is reflected in the spiking activity of the neuron, with no adaptation and identical spiking activity as the classical model when 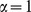 and increasing spike adaptation as 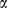 decreases (Fig. 1D). Our model shows an inverted dependency of the latency of the first-spike to the value of 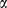 (Fig. 1E). The first-spike latency is common in the activity of neurons and has been suggested as a source of information for decision accuracy [48]–[50]. The ISI of the model in response to constant stimulation shows increasing adaptation as the value of 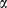 decreases. An analysis of the ISI responses shows that for values 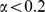 the slope of the ISI follows a power-law distribution(Fig. 1F). Power law distribution of ISIs may maximize firing rate entropy [51]. It is important to emphasize that this variability arises from the intrinsic properties of the model since the input is constant. As a consequence our fractional order model produces spike trains with a wide range of inter-spike intervals, with power-law dynamics for smaller values of 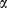 (Fig. 1G).

**Figure 2:**
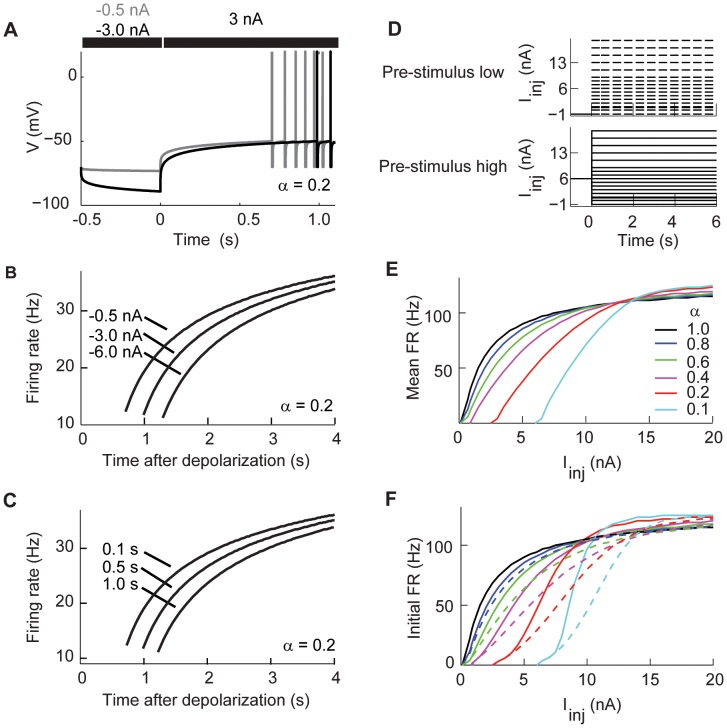
The mean and instantaneous firing rate responses of the fractional model to constant input. (A) Two levels of hyper-polarizing current followed by the same depolarizing current result in different spiking patterns. (B) The instantaneous firing rate against time for different conditions described in A. (C) The instantaneous firing rates against time for identical hyper-polarizing current (−3 nA) with different durations. (D–F) Comparison of mean and instantaneous firing rates. (D) Top: Applying a hyper-polarizing (pre-stimulus low, dashed lines) current before application of current steps to calculate firing rate responses. Bottom: as in the Top but applying a depolarizing (pre-stimulus high, solid lines) current. (E) The mean firing rates (mean FR) vs injected current show Type I response for both (pre-stimulus low) and (pre-stimulus high) current input paradigms described in D. The dashed and solid lines corresponding to pre-stimulus low and high, respectively, overlap. (F) The instantaneous firing rate to the stimulations described in D calculated from the first inter-spike interval depends on past activities. Dashed lines correspond to pre-stimulus low paradigm, solid lines correspond to pre-stimulus high.

The formulation of the fractional leaky integrate-and-fire model and the results in Fig. 1 suggest that the activity of the neuron depends on the integration of previous activity beyond the value of 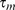. If the neuronal dynamics depend on the memory trace then the amplitude and time of a previous input would affect the spiking activity of the fractional model. To determine this point we injected two different amounts of a hyper-polarizing current for a fixed period followed by an identical depolarizing current (Fig. 2A). As is well known, the firing rate response of classical leaky integrate-and-fire model shows basically identical responses except for the time to first spike after the onset of the depolarization. However, the fractional model suggests a more complex response in which not only the time of the first spike but the instantaneous firing rate of the ensuing spiking response depends on the value of the hyper-polarizing current (Fig. 2B). Similar results are obtained when there is a fixed hyper-polarizing current applied for different amounts of time. In this case, the longer the fractional model integrates the hyper-depolarizing stimulus, the more it affects the delay to first spike and spike adaptation (Fig. 2C).

Since the response of the fractional model is history dependent, the firing rate curve versus injected current might change depending on previous input. To test this possibility we injected either a hyper-polarizing (pre-stimulus low, Fig. 2D top, dashed lines) or depolarizing (pre-stimulus high, Fig. 2D top, solid lines) current to the model before injecting current for 6 s to calculate the firing rate. We calculated the mean firing rate of the spike train produced during the last 3 s of the stimulation. This quantification of the firing rate shows that as 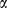 decreases the threshold to generate spiking activity increases. However, the firing rate curves resulted in the same value independent of the previous stimulation (Fig. 2E, the dashed lines for the pre-stimulus low and solid lines for the pre-stimulus high overlap), thus showing that after 3 s the fractional model reaches steady state while showing a typical Type I dependency [52], [53]. Although the steady state response of the system is independent of past activity, the initial instantaneous firing rate of the first inter-spike interval shows dependence on previous activity [54]. For 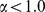, the initial firing rate calculated from (pre-stimulus high) (Fig. 2F, solid) is always higher than the initial firing rate calculated from (pre-stimulus low) (Fig. 2F, dashed). Thus, this analysis shows that although the model is integrating all the voltage-memory trace the influence of this component decreases over time.

#### Adaptation to periodic inputs

**Figure 3:**
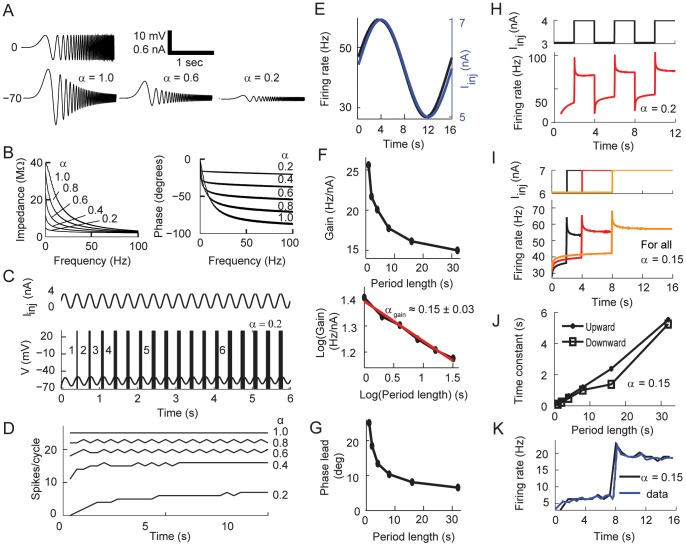
Sub-threshold and spiking fractional dynamics to oscillatory inputs. (A–B) Voltage responses, impedances, and phase angles of the fractional order model in response to a ZAP current. (A) A time varying sub-threshold current input (Top) and the voltage responses for three different values of 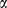 (Bottom). (B) Impedance and phase analysis for the simulations in A as a function of 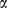. (C–D) Spiking response to just above threshold sinusoidal input (C, Top). The neuron generates an increasing number of spikes per cycle (C, Bottom). (D) The number of spikes per cycle for identical input as in C and varying 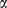. (E–G) Response to supra-threshold sinusoidal input. (E) The fractional model with 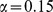 instantaneous firing rate (black) in response to a sine wave input (blue). (F) The gain of the firing rate with respect to the period length of the input (Top) shows power-law dynamics when plotted in log-log (Bottom). The slope of the best-fit line (red) for the log-log gain curve is -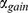. (G) The phase lead of the firing rate in response to the same sine wave current. (H–J) Response to square periodic input. (H) In response to square wave current (Top), the fractional model displays upward and downward spike rate adaptation for 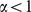. (I) The instantaneous firing rate shows upward and downward adaptations in response to changes in the period of the square input (4, 8 and 16 s). (J) The time constants of both upward and downward adaptations in (I) increase when the period of the alternating input current increases. (K) The spike rate adaptation of L2/3 neocortical pyramidal neurons with period 16 s (Fig. 1C in [37]) is fitted with the spike rate adaptation of the fractional model with 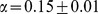 (95% confidence interval) using least-squares fitting. The alternating input current is switched between 3.4 and 4 nA. For E–K we used 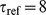 ms and 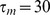 ms to better replicate the experimental data.

We decided to systematically study the response properties of the fractional model to oscillatory input from the sub-threshold to the spiking regime. A common quantification method of the sub-threshold properties of a neuron is to use a frequency varying sinusoidal current, also known as a ZAP current (Fig. 3A top). As is well known [55], [56], the amplitude of the sub-threshold voltage oscillations of the classical model decreases as the input current frequency increases. This is a consequence of the low-pass properties of the classical leaky integrator. Our model replicates this behavior for 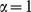. However, the voltage response decreases in amplitude as 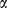 decreases (Fig. 3A). We quantified the input-output relationship of the model by calculating the impedance magnitude in the frequency domain (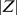) [57], [58] (see Methods). As expected for the passive membrane model, the peak impedance is at the lowest frequency and there is no resonance for 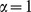. This property of the model is robust and not affected even for smaller values of 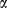. However, for smaller values of 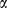 the impedance becomes almost constant across all tested frequencies (Fig. 3B left). We also analyzed the phase shifts between the input current and the output voltage. The phases are always negative indicating lags in which the voltage oscillation follows the input current. However, the phase angle becomes less negative when 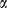 decreases indicating smaller lags which are practically the same across all frequencies (Fig. 3B right). Overall, our results show that lower values of 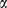 result in a low but homogeneous filter of oscillatory inputs across all frequencies.

In order to initially characterize the spiking response to an oscillatory input we used a sinusoidal current with constant frequency and amplitude (Fig. 3C). We chose parameters that generated supra-threshold spiking at the peak of the oscillation and no firing at the trough for 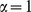. As expected, as the value of 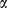 decreases the response of the neuron is delayed. Interestingly, this results in a spiking response to the oscillatory input in which the number of spikes per cycle increases slowly. This behavior is due to the accumulation of the input in the memory trace of the fractional model (Fig. 3D). We then characterized the response of the neuron to supra-threshold oscillatory input (firing at any value of the input current) while varying the period of the stimulation. For this purpose we calculated the gain of the spiking activity of a neuron, which is defined as the ratio of the amplitude of the instantaneous firing rate to the amplitude of a sinusoidal input current with a fixed period length. Experimental results have shown that the firing rate of L2/3 pyramidal neurons behaves as a fractional differentiator [37] in which the firing rate follows the fractional derivative of the input. In this case, it is 
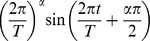
 when the input is 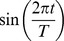 with period 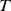. We simulated such an experiment in our model, with 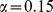 (Fig. 3E). We then fitted the resulting instantaneous firing rate with a sine wave in which the free parameters were the amplitude and the phase. Our results show, as demonstrated experimentally, that the gain decreases as a function of the period following a power-law [37]. A least-square fit of these data points shows 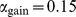, which matches the order of the fractional leaky integrate-and-fire model used in the simulation (Fig. 3F). Although it has been reported that the phase lead is frequency independent [37], our results show that the phase lead decreases as the period of the sine wave increases (Fig. 3G), which is consistent with more recent reports [59]. The gain power-law behavior and phase lead response are preserved even in the presence of noise added to the sine wave input (not shown), again, replicating experimental results. This analysis shows that the fractional sub-threshold voltage behavior of the model is reflected in the spiking activity of the neuron.

There is an increasing body of evidence showing that when a cortical neuron is stimulated with alternating current steps the spiking activity of the neuron shows adaptation dependent on the period of the stimulus [37]–[40]. Our model reproduces this dynamic, particularly when 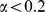 (Fig. 3). As done in the previous paragraph we calculated the instantaneous firing rate of the model when delivering a supra-threshold alternating square current. As expected, when 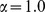 the spike frequency shows adaptation only due to the membrane time constant (not shown). As shown experimentally and for values of 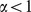, when the input current makes a transition from a low input state to a high input state the spiking activity increases and then relaxes over time to a lower firing rate. We call this downward spike adaptation. Similarly, when the input goes from a high to low state the firing rate decreases and then adapts back to a higher firing rate. We call this upward spike adaptation (Fig. 3H). As done experimentally, we fitted a single exponential to the downward or upward adaptation responses. The results show that the time constants of the upward and downward adaptation are constant across the multiple periods of the input signal when the signal has a fixed period (not shown). However, again as shown experimentally, the time constant of adaptation is a function of the period of the input (Fig. 3I and J) [37]. Our model further replicates the firing rate adaptation reported by the same group for neurons receiving an input of period 16 s (Fig. 1C in [37]). In their report the authors calculated an average value of 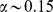 which is well fitted by our model using the same value obtained by a least-squares fit (Fig. 3K). Our modeling results indicate that the fractional order of the derivative is less than 0.2 in order to replicate experimental results, which is in good agreement with the fractional exponent determined experimentally [37].

#### History dependent spike adaptation

**Figure 4:**
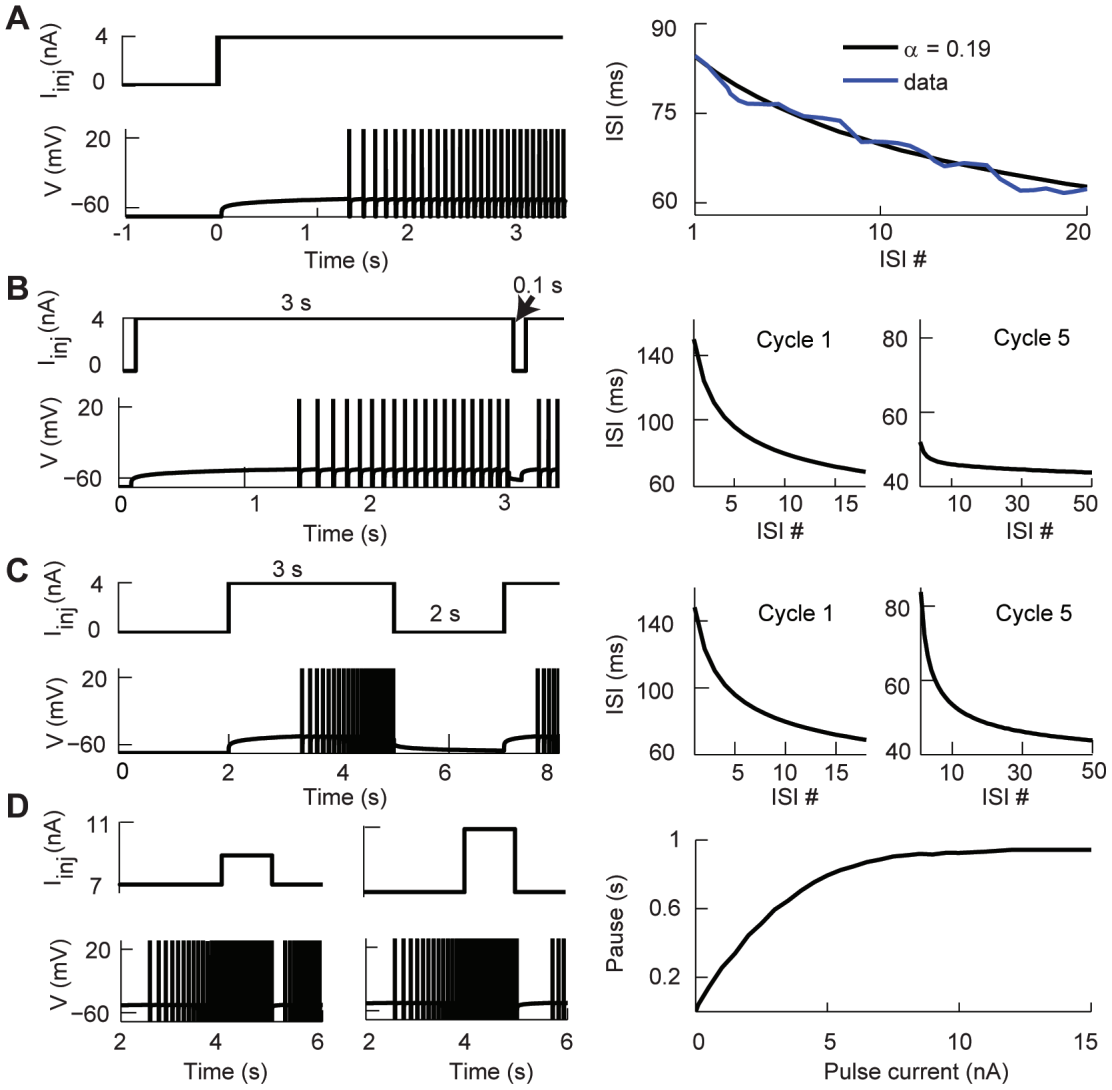
Inter-spike interval adaptation and history dependence. (A) Left: The spiking activity of the fractional model with a step current of 4 nA. Right: The inter-spike interval (ISI) curve of Layer 5 pyramidal neurons in primary motor cortex (Fig. 2B in [38]) is fitted with the ISI curve of the fractional model with 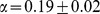 (95% confidence interval with a least-squares fit). The first 7 ISIs of the model are removed for best fit. (B–C) Modeling the intrinsic memory of adapting pyramidal cells. (B) Left: The voltage trace of the model in response to a step current separated by 0.1 s inter-stimulus interval. Right: The ISIs of Cycle 1 and 5 as a function of ISI number. (C) The same as panel B, but with longer inter-stimulus interval, 2 s. For A–C 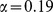. (D) Memory induced pauses of the model with 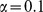 depend on the magnitude of the current pulse. Left: Voltage traces with shorter and longer pauses in response to 1 nA and 4 nA current pulses, respectively. Right: The pause of the spiking activity increases as a function of the magnitude of the current pulse. For all we used 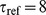 ms and 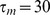 ms to better replicate the experimental data.

In this section we analyze the adaptation and history dependence of the ISI. The spiking dynamics are affected by the long-range dependence of the voltage dynamics and the intrinsic membrane conductances. Both long-term and short-term intrinsic memory traces are observed in pyramidal neurons [37], [38], [59]. It has been recently found that the spiking activity of some Layer 5 pyramidal neurons in primary motor cortex has a long first spike latency and its ISIs decrease over time [38]. Using standard parameters we varied the value of 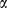 when stimulating the model with a step current (Fig. 4A left). We then fitted the plot of the ISI versus the ISI number to the data reported by Miller et al. (Fig. 2B in [38]). We found that when 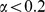 the dynamics of the ISI can be reproduced (Fig. 4A right). The same authors studied adaptation by applying a supra-threshold current for five cycles separated by an inter-stimulus interval (gap) [38]. We implemented this protocol in our model and compared the dynamics of the inter-spike intervals during Cycle 1 and Cycle 5. When the gap is as short as 100 ms, our results show that there is spike adaptation during Cycle 1 but very little in Cycle 5 (Fig. 4B). In contrast, when the gap is increased to longer durations the spiking dynamics of Cycle 5 is very similar to that of Cycle 1 (Fig. 4C). We calculated the ratio of the second-to-last ISI in Cycles 1 and 5 to quantify spike adaptation as a function of the gap. Using this metric Miller et al. found that total recovery required a gap of 1 s (their Fig. 7C [38]); in our case the recovery takes about 1.5 s. However, absolute recovery of the first ISI between Cycles 1 and 5 took longer than 25 s (not shown). Similar to spike adaptation to constant input, neurons respond with a silent period after strong stimulation [18]. We replicated this phenomenon by decreasing 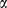 to 0.1 and injecting a square current with different amplitudes on top of a constant supra-threshold input for a fixed period of time (Fig. 4D left). After the square input was turned off there was a pause in the spiking activity that depended on the input strength (Fig. 4D right). Of course, since the maximum firing rate is bounded by the refractory period then the system reaches a limit (around 950 ms and 9 nA). This qualitative behavior of the fractional model is in good agreement with the behavior of the integrate-and-fire model with adaptation current (Fig. 5 in [18]), and with the firing behavior observed experimentally in rat subthalamic neurons (Fig. 7 in [60]). Thus, there is a slowly decaying memory trace that can affect the ISI adaptation for very long periods of time. Our analyses suggest that all these different neurons might be fractional differentiators.

**Figure 5:**
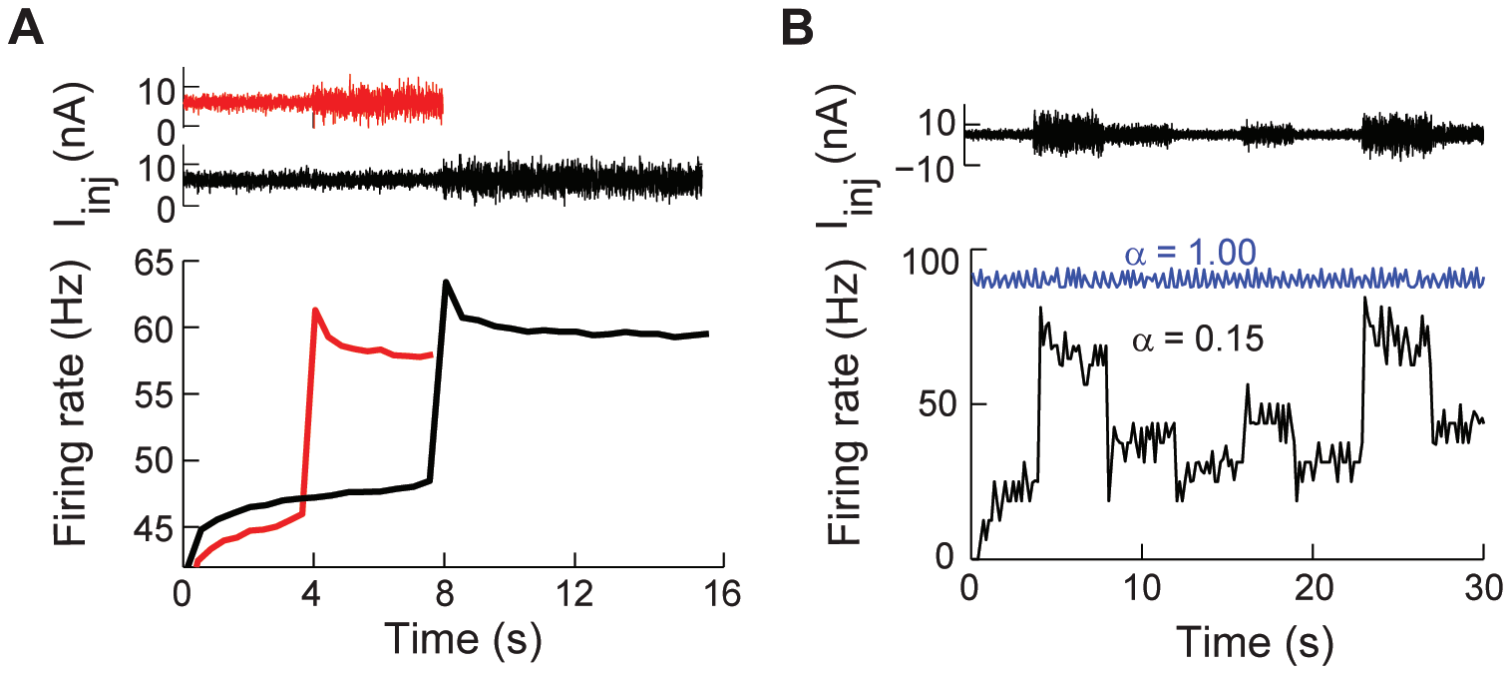
Firing rate adaptation to changes in input variance. (A) Top: Noisy input current with two standard deviations: 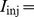
6+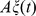 nA, 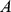 = 1 or 2 nA. Bottom: The firing rate to noisy input current calculated from 100 trials, 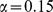. (B) Top: Time varying noisy input current. 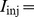
5+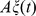 nA, 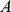 = 1, 4, 2, 1, 2, 1, 4 and 2 nA, consecutively, and the noise 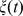 is filtered with an alpha function 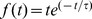 with 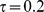 ms. Bottom: Instantaneous firing rate in response to the input current for 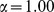 (blue) and 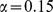 (black). For both 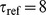 ms and 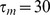 ms.

**Figure 7:**
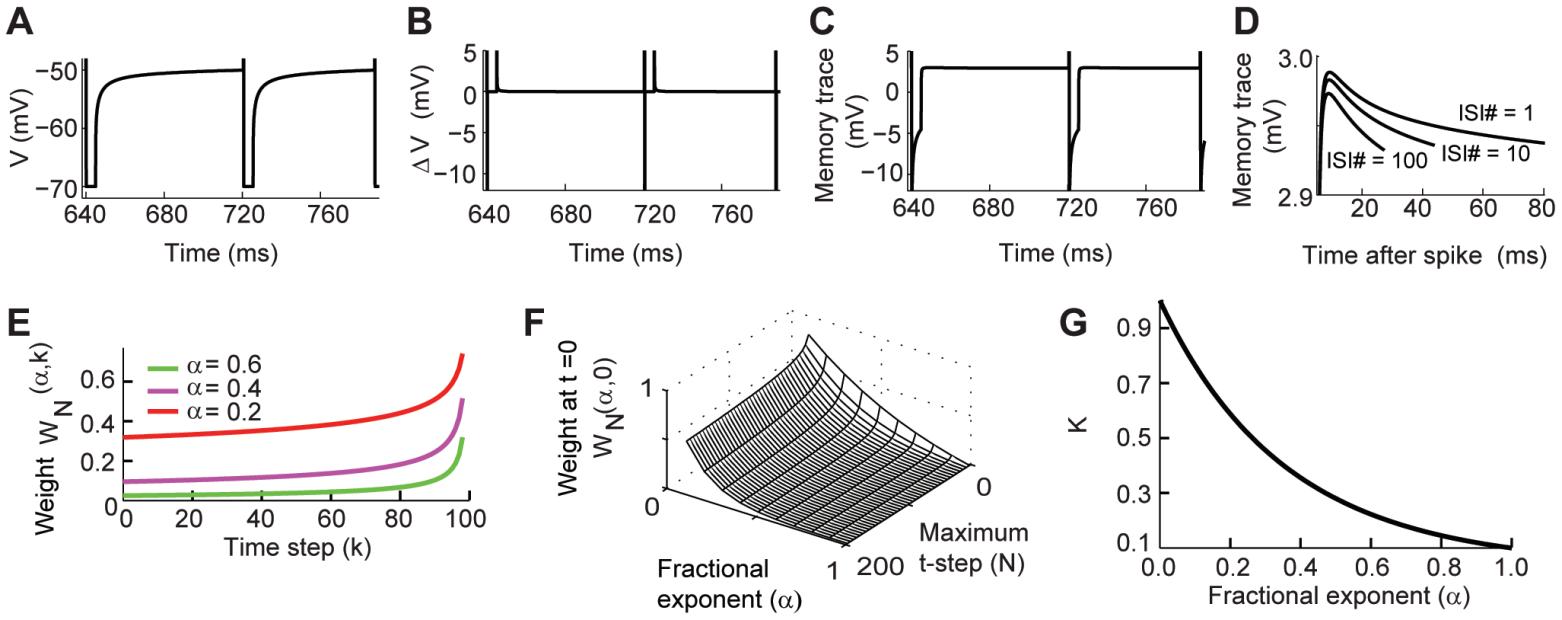
The properties of the voltage-memory trace. (A–D)The changing response of the memory trace across multiple spikes, 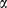 = 0.2. (A) Voltage trace of the fractional model stimulated with 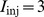 nA. Spikes have been clipped to emphasize the sub-threshold dynamics. (B) 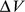 for the data in A. (C) The memory trace for the data in A. (D) Overlapped memory traces for different inter-spike intervals during the same simulation. (E–G) The dynamics of the weight of the voltage-memory trace 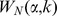 and the fractional coefficient depend on the fractional exponent 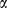. (E) When 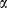 decreases the weights increase. (F) The value of the weight 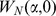 as a function of 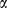 and time. (G) The fractional coefficient of the Markov process 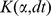 increases when 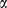 is decreased.

#### Spike rate adaptation to changes in the variance of noisy input

The adapting behavior of neurons also depends on the variance of the noisy input current [37], [61]. We injected a noisy current with zero mean and varying standard deviation to the fractional model with 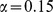 (Fig. 5A top). Our results show that the spiking activity of the neuron has upward and downward firing rate adaptation (Fig. 5A bottom) (compare with Fig. 1D in [37] and Fig. 3A in [61]). As in the case of the oscillatory noiseless input, the adaptation time constant increases directly proportional to the period of the input (not shown). This property allows the model to follow changes in input variance as opposed to the classical leaky integrate-and-fire model (Fig. 5B). Changing the variance of the input noise can affect the spike rate of the classical integrate and fire model only when the mean current is very close to the threshold current (not shown). Thus, the fractional model can reproduce variance-dependent adapting behaviors such as the ones observed in L2/3 neocortical pyramidal neurons (Fig. 5B in [37]) and barrel cortex neurons (Fig. 2 in [62]).

#### Spike-time reliability of the fractional integrate-and-fire model

**Figure 6:**
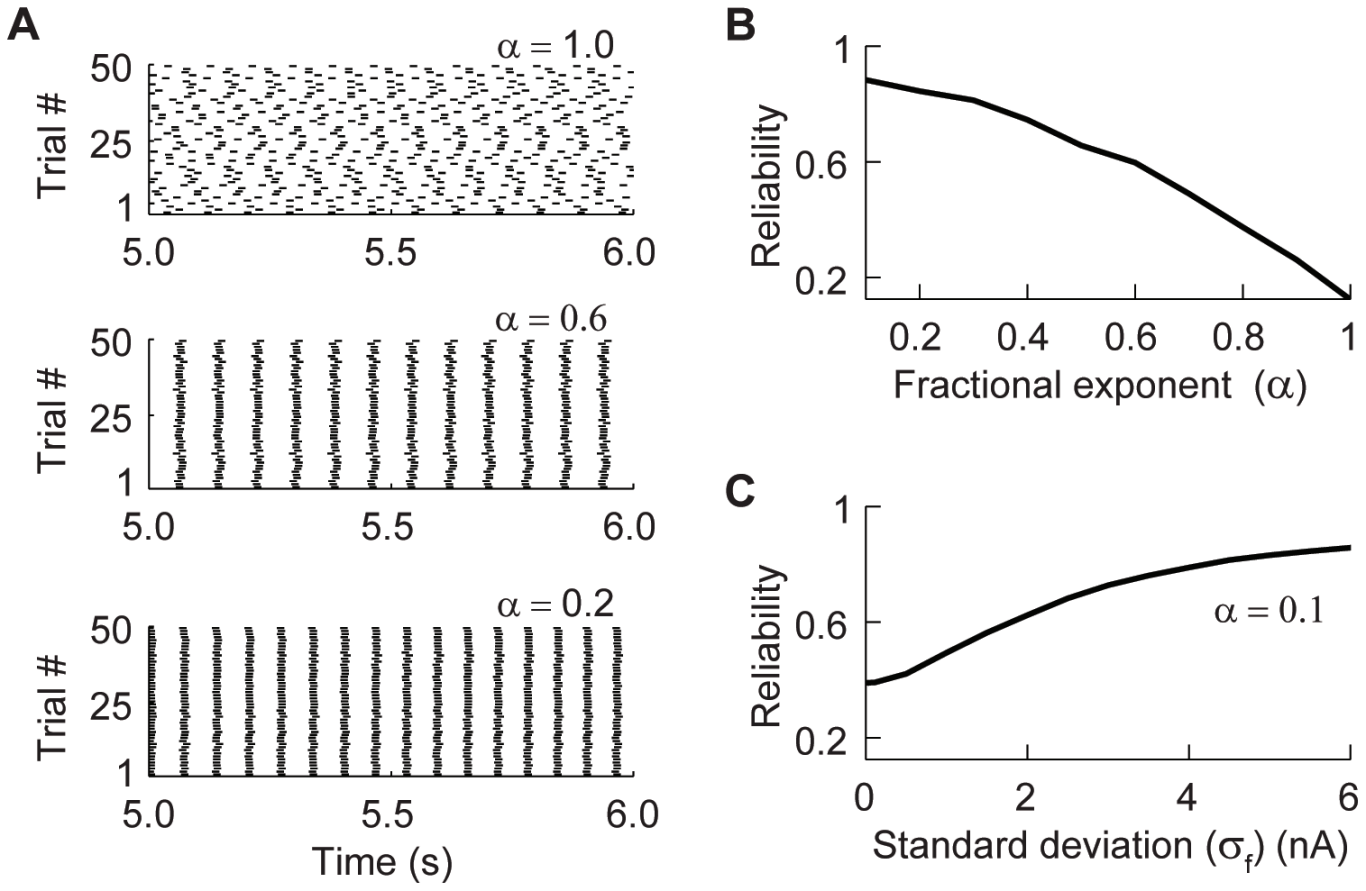
Spike-time reliability increases as the fractional exponent 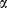 decreases. (A) Raster plots of the response of the fractional model to a noisy input under three different values of 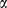. (B) Spike-time reliability of the fractional model increases as the fractional exponent 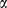 decreases. (C) Reliability increases when the standard deviation of an embedded fixed signal increases. See Text and Methods for details.

Unlike constant inputs, stochastically fluctuating inputs can generate spike trains with highly reliable spike timing across repetitions [63]–[66]. The reliability of spike patterns is influenced by the mean, variance, and frequency of the stochastic input [67]–[69]. To test the reliability of the fractional model we again injected a constant current with added Gaussian noise. The value of the current was chosen for each 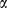 so that the model produced low firing rate for all values of 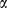 (average 14 spikes/s). A raster plot analysis of our results shows that the inter-trial variability decreases as 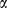 decreases (Fig. 6A). We quantified this reliability using a widely used correlation-based measure [68]–[71] (see Methods). This analysis shows that the spike reliability increases as the fractional exponent 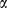 decreases (Fig. 6B). The reliability of a neuron also increases when the variance of a time-varying signal embedded in a noisy signal increases [63], [64], [69], [72]. We added a fixed noisy signal 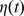 to the injected current and variable noise to analyze reliability for 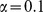. The results show that the spike-time reliability depends on the variance of the embedded signal (Fig. 6C) [63], [64], [69], [72]. Taken with our previous results, our analysis shows that when 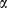 is decreased the fractional model produces spike trains with strong spike time adaptation and high spike reliability.

### The properties of the fractional derivative that provide a memory trace to spiking dynamics

The voltage memory provides non-local dynamics that affects the spiking activity of the cell. The voltage-memory trace decays over time and is dependent on the value of 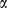. For 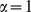 the process is identical to the classical leaky integrator, while for values of 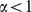 the past trajectory activity increasingly contributes to the present value of the voltage. Since the weight 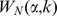 is always positive, the sign of the voltage-memory trace depends on 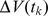. With positive applied current the fractional model generates action potentials (Fig. 7A), with 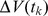 positive until the cell fires a spike and is reset (Fig. 7B). However, when there is a spike and voltage is reset, 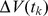 becomes negative. After the voltage escapes from the refractory period, the voltage-memory trace is positive until the next spike (Fig. 7C). As opposed to the classical leaky integrate-and-fire model, the memory trace accumulates over multiple spiking events, changes its dynamics, and thus it affects the ISI (Fig. 7D).

The weight of the voltage-memory trace 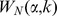 is determined by the fractional order 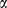. The weight 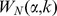 is 0 for 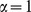 and it is always positive for 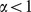. Fig. 7E shows the results of a simulation with standard parameters for 100 time steps (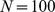). The x-axis corresponds to the 1–100 temporal weights at t = N. The y-axis corresponds to the value of each weight 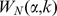. The increase in weight can be interpreted as the influence of the past state on the future state of the voltage. In a classical leaky integrator any past value is forgotten as a function of the time constant. In the fractional leaky integrator all the past values could continue to influence the future behavior of the system, particularly, for low values of 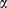. Fig. 7F illustrates this point by showing the dynamics of the weight of the initial condition (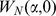) as a function of 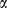. The other important term that comes from the fractional derivative is the fractional coefficient 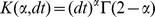 which is a weight factor for the Markov process (Eq. 6). When 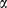 becomes smaller, this function grows rapidly and it makes the effect of the input current on the voltage dynamics stronger (Fig. 7G). It is the combination of the weighted Markov process and the opposite effect of the memory trace that contribute to the long term spiking dynamics of the fractional model.

**Figure 8:**
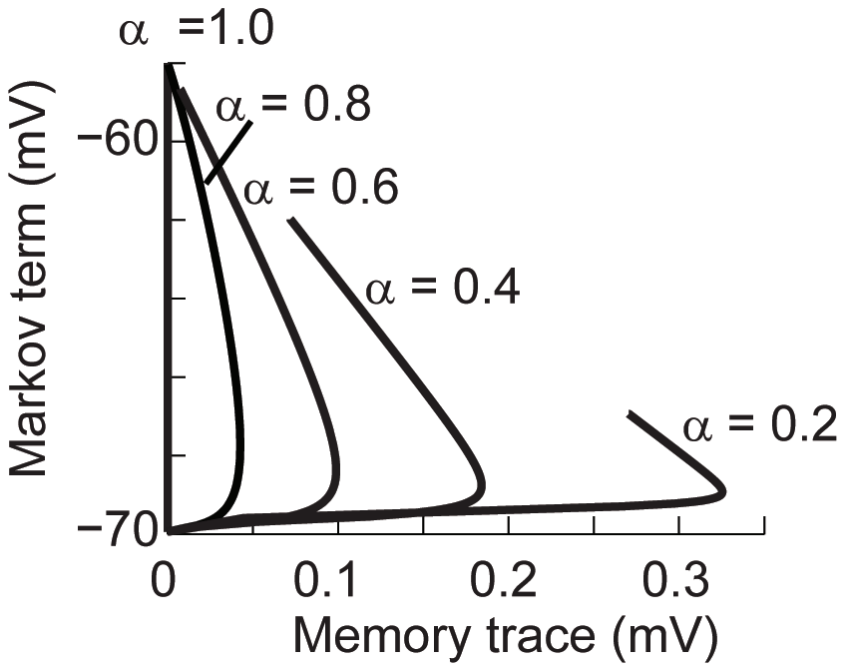
The memory trace dominates the fractional dynamics for low values of 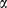. Markov term versus memory trace as a function of 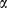. The fractional model was stimulated with constant current (0.3 nA) for 5 seconds. For 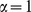 the memory trace is zero and the voltage only moves along the y-axis.

Most of our results suggest that the value of 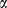 has to be low in order to reproduce the spike timing adaptation observed experimentally. Although the fractional model has a continuous dependence on 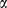 the power-law dynamics cause the effects to be nonlinear. For 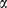 close to 1 the effects of the Markov term weighted by the gamma function dominate the dynamics. It is only when 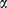 decreases that the voltage-memory trace can slow down the time evolution of the voltage. This is illustrated by plotting the value of the Markov term versus the memory trace for simulations in which we apply a step current (Fig. 8). When 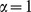 the memory trace is zero and the voltage only moves along the Markov term axis. As the value of 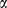 decreases the voltage trajectory is deflected, taking longer to depolarize. The power-law dependency results that when the value of 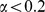 in that the memory trace dominates in the initial moments of the depolarization and slows down the dynamics (Fig. 8).

### Comparison to other models of fractional integrate-and-fire

**Figure 9:**
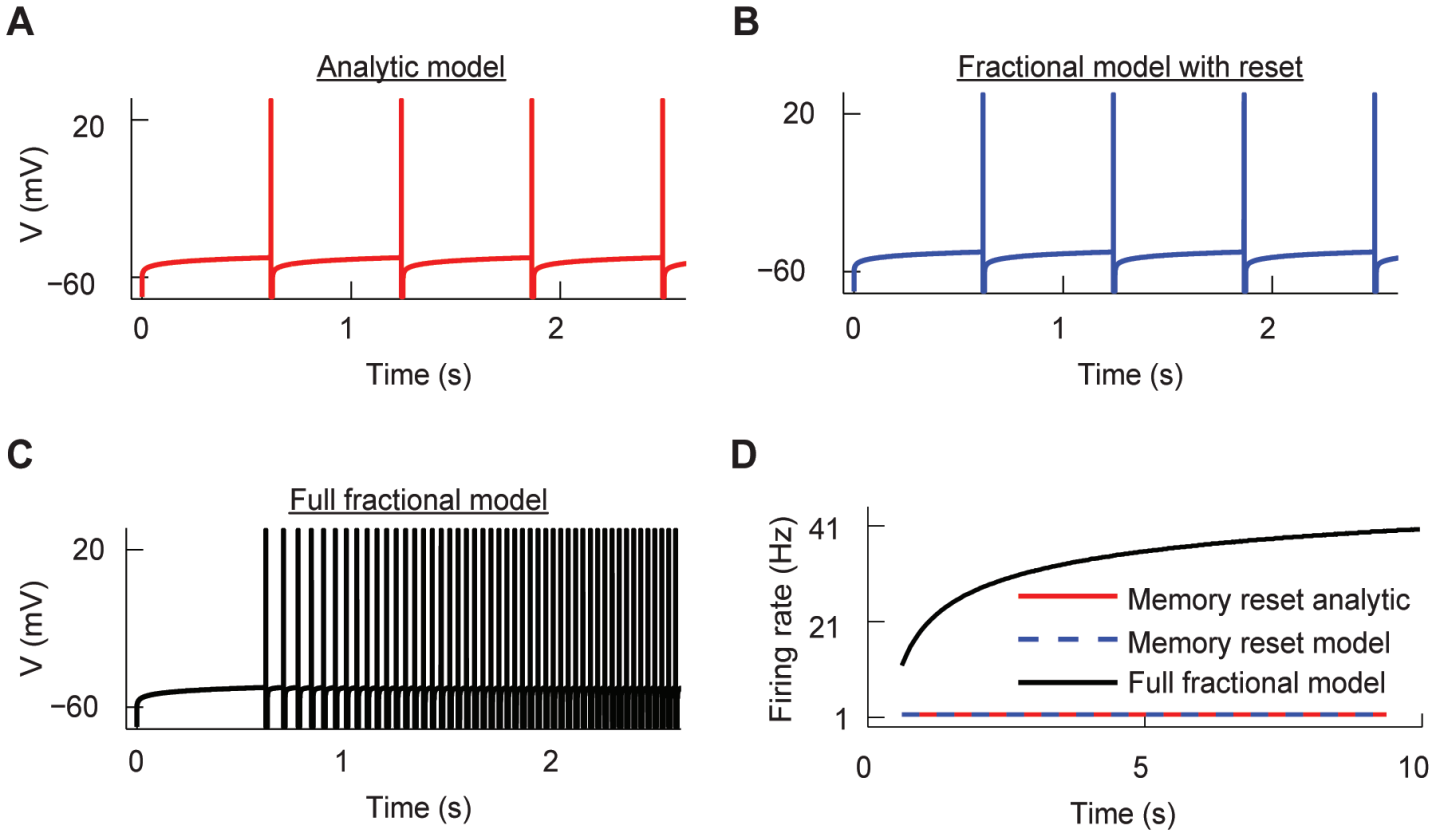
The fractional model and its analytic solution with memory reset show no spike adaptation. (A) The spike train produced by the analytic solution with memory reset displays regular spiking. (B) The spike train produced with the fractional model with memory reset also displays regular spiking. (C) The spike train produced by the full fractional model without any memory reset display spike adaptation. (D). The firing rate curves of the analytic solution, fractional model with memory reset and full fractional model. For all panels 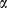 = 0.1, 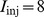 nA, 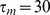 ms and 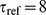 ms.

When the input current is constant and 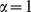, the sub-threshold voltage dynamics (Eq. 1) have an analytic solution which is the same as the solution of the classical integrate-and-fire model [73]. Similarly, for constant input current Langlands et al. [74] derived the analytic solution of the sub-threshold voltage for 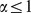 from the fractional cable equation. In that model the integration of the memory trace is restarted after every spike, thus wiping out the memory trace (see Methods). Such a system is capable of reproducing the delay to first-spike to constant input but then produces regular spiking (Fig. 9A). Our fractional model replicates the same result when we reset the memory trace after every spike (Fig. 9B). However, our model greatly differs from the analytical solution when taking into account the cumulative effect of the memory trace across multiple spiking cycles (Fig. 9C–D). Although both the analytic and full model with memory reset can capture short term memory, they do not produce spike adaptation. Hence, the full fractional model without any memory reset captures the multi-scale processes that spans the spiking activity of neurons.

## Discussion

Spike timing adaptation is a widespread phenomenon throughout the nervous system [37], [39], [75]. In particular, neocortical pyramidal cells produce spike adaptation with multiple timescale dynamics [5], [37], [59]. Our model is capable of reproducing multiple sub-threshold and spike timing adaptation properties reported by different groups and with different experimental conditions. The conclusion from fitting our model to experimental results is that 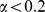. This indicates that the order of the fractional derivative has to be very low for the memory trace to overcome the classical contribution of the leaky integrator. Furthermore, the fractional model is capable of producing spike trains with high adaption and reliability. Our work provides a framework to study spike adaptation as part of power-law dynamics and the techniques used here can be applied experimentally to determine if a neuron is following power-law adaptation from the sub-threshold to firing rate regimes.

### Using fractional dynamics to study non-local interactions

The fractional model can produce different degrees of adapting electrical activities by modifying the fractional exponent 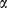. A fractional order derivative captures the long-range correlations of the a system models that results in non-local dynamics. Fractional differential equations have been used in biological systems to capture the long-term memory effects of the dynamics [33], [74], [76]–[81]. For example, fractional order derivatives have been observed in the vestibular-ocular system [27], [82] and in the gating dynamics of ion channels [44], [83]. Power law statistical distributions of a neuronal response also exhibit fractional order dynamics [37]. The voltage dynamics in the fractional order model depend on both the Markov term (immediate past) and the voltage-memory trace that integrates all past voltage values. The behavior of the voltage-memory trace is similar to the behavior of the adaptive filter in the work of Pozzorini et al. [59], although in their work this filter is described as the sum of the spike-triggered current and a moving threshold. The voltage-memory trace also corresponds to the adaptation integral used in other works [18]. In this context our fractional order leaky integrate-and-fire model is a unified mathematical and computational framework that can be used to describe power-law dynamics and long-range correlations in neuronal activity. The fractional derivative can capture relationships between the distribution of conductances that can be complicated to model using explicit techniques.

### The biophysical interpretation of the fractional order of a neuron

In biophysical systems, the voltage-memory trace might represent spike-triggered mechanisms that cause adaptation. For example, the voltage-memory trace might represent the slowly inactivating potassium-like current that induces the upward spike adaptation shown in Layer V pyramidal neurons of primary motor cortex [38], suggesting that these neurons are fractional differentiators. Alternatively, the voltage-memory trace can correspond to other adaptation currents such as calcium-activated after-hyperpolarization currents [5], slow sodium-channel inactivation currents [84], [85], or a combination of several adaptation currents. Many studies have used models with slow adaptation currents and exponential functions to analyze spike time adaptation [5], [54], [84]. Some of these models can produce similar properties found in fractional dynamics. For example, the Hodgkin-Huxley model with slow after hyper-polarization currents can produce multiple timescale adaptation processes [37]. The Generalized leaky integrate-and-fire model with an adaptive filter (GLIF-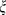) produces spike adaptation with power-law dynamics [59]. Both the power-law dynamics and history-dependent properties of the GLIF-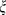 model correspond to that of the fractional leaky integrate-and-fire model. However, the fractional model provides a general way by simplifying the complicated details shown in other models. The fractional model exhibits spike adaptation with power-law dynamics by integrating all the past voltage values without any additional adaptation currents. Power law functions generalize the mechanism underlying exponential processes and are better alternatives to describe scale invariant spike adaptation [17], [18], [42], [59], [86], [87]. The fractional model shows new directions for studying spike adaptation using fractional derivatives and power-law dynamics instead of classical derivatives and exponential functions.

The key parameter in our fractional model is 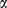. Experimental results have suggested that 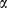 can be as small as 0.15 for neocortical pyramidal neurons [37]. Our fitting of these data also resulted in a value of 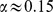. Fits to the response of Layer 5 pyramidal motor cortex neurons resulted in a value of 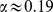 [38]. Thus, the experimental results and our modeling analysis suggest that the biophysically important values of 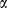 are when 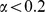. However, it is clear that if 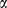 is much closer to 0, the system takes a longer time to generate spikes or never fires spikes at all, depending on the magnitude of the stimulus, so the feasible range of the fractional exponent might be between 0.05 and 0.2. The value of 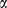 might correspond to the type, function, or location of specific neurons [38]. These regional differences are not exclusive to the cortex, for example, Purkinje cells in Lobule X and Lobules III – V show different degrees of spike adaptation [75]. Thus, different values of 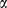 can be used to map the general voltage and spike time adaptation properties of neurons throughout the brain.

### Determining if a neuron is a fractional differentiator

Previous work has provided an experimental foundation to determine if a spiking neuron is a fractional differentiator [37]. Our framework provides a more general methodology to determine power-law neuronal dynamics from the sub-threshold to the spiking regime. At the sub-threshold level there are several measurements that can indicate that the membrane of the neuron follows a power-law. For example:

- The flattening of the impedance and phase response to the ZAP current.
- A power-law behavior can be measured by applying a low depolarizing current and plotting Log(voltage/time) versus Log(time). A straight oblique line indicates power-law behavior.

In the same experiment a series of protocols can also be applied to determine if the spiking activity follows power-law dynamics. Some of these measurements are straightforward, others require longer recordings. For instance:

- The ISI histogram plotted as Log(Counts) versus Log(ISI) follows a power-law.
- The instantaneous firing rate response to step currents depends on the value of the current before the stimulation.
- Using a sinusoidal or square oscillatory input with varying period, fit a time constant to the adapting firing rate. If the time constant depends on the stimulus this suggests a memory trace.
- The gain of the spiking neuron follows a power-law.
- The ISI adaptation depends on the inter-cycle (gap) time and the pause length of the neuron depends on the strength of the previous stimulus.
- The neuron shows firing rate adaptation to changes in variance with fixed mean.

### The computational importance of power-law spike time adaptation

Although power-laws are found at multiple scales of biological organization, their function and importance are still debated [88], [89]. In our work we propose that the membrane voltage of neurons can follow a power-law due to the emergent property of the combination of multiple active conductances. The value of the fractional derivative can be mapped to spike time adaptation dynamics taking place in multiple cell types across the brain. Computationally, a low value of 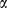 results in spiking dynamics that are at the same time highly adaptable and reliable. Thus, neurons following power-law adaptation could have a large operational range while providing the reliability to filter out noisy signals and increase information capacity. The lack of a sub-threshold resonance frequency allows the neuron to filter signals homogeneously over a wide range of frequencies. In such a case, the fractional leaky integrate-and-fire model provides the basis to study the computational capacities and information processing properties of neurons showing high degree of spike time adaptation.

## Methods

### Implementing the fractional leaky integrate-and-fire model

The equations were coded and implemented using our recently developed fractional integration toolbox [47] and the simulation software package MATLAB [90]. The toolbox can be downloaded at www.utsa.edu/SantamariaLab. The parameters for all simulations were fixed and are described in Table 1.

### Comparing models to experiments

In order to compare to experiments we extracted the data from the referenced material using WebPlotDigitizer (http://arohatgi.info/WebPlotDigitizer/). Then we imported the data points into Matlab. We then ran simulations varying the value of 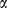, usually between [0.5, 1.0] at 0.1 steps. We minimized the mean squared error between the data and the simulations, 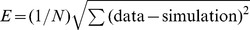, where 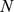 is the number of points. We determined the 95% confidence intervals by then varying 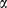 around this minimum value of 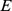 and calculated when it changed for an amount larger than 5% in either direction.

### Modeling oscillatory input

In order to get only sub-threshold oscillations we used 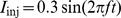 where 
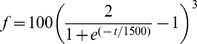
 Hz is a sigmoidal frequency function of time that varies from 0 Hz to 100 Hz in 10 seconds. The impedance as a function of frequency is defined as 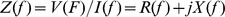, where V is the membrane voltage, I is the input current, R is the resistance and X is the reactance. The absolute value of 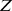 can be calculated using a fast Fourier transform 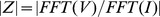 and the phase as 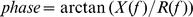 [57].

### Reliability quantification

The simulations to study spike time reliability were generated by injecting a current 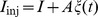 nA, where 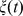 is a Gaussian white noise with zero mean and standard deviation 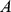 = 0.03 nA. The stochastic input 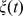 is filtered with an alpha function 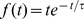 with time constant 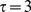 ms. The spike trains were obtained from 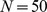 trials, and the trail-to-trail variability of those N different responses were caused by the noise 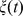 while 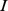 and 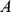 were fixed [68], [71]. In order to avoid initial condition effects we analyzed the spike trains of the last 5 s from 10 s simulations.

The reliability measurements were computing using a correlation-based measure [68]–[71]. In brief, the spike trains obtained from N trails were smoothed with a Gaussian filter of width 3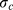, and then pairwise correlated. The correlation-based measure reliability 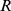 is defined as
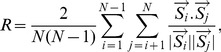
(10)where 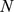 is the number of trials and the vectors 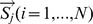 are the filtered spike trains, filtered using 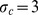 ms. The values of 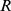 range from 0 (lowest reliability) to 1 (highest reliability), and the reliability 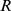 was computed for 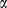 in the range [0.1, 1.0].

For the quantification of the reliability in the coding of an embedded signal we injected the following current 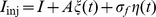 nA, where 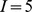 nA is the mean current which generates very low firing rate, 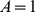 nA is the standard deviation of the intrinsic noise, and 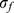 is the standard deviation of the embedded signal also generated by a Gaussian noise and varies from 0 to 6 nA. The intrinsic and embedded signals are filtered with an alpha function 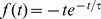 where 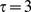 ms.

### The comparison of the analytical and full fractional model

We compared our fractional model to a previously developed analytical model of a fractional leaky integrate-and-fire [74]. Briefly, this analytical model is obtained with the following steps. Equation 1 can be converted to
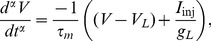
(11)where 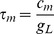 is the membrane time constant. By applying 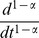 on both sides we obtained
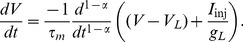
(12)
Equation 12 is solved using the Fourier-Laplace transform (for details see [36], [74]) and the solution is given by
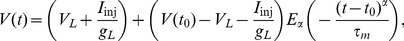
(13)where 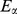 is the Mittag-Leffler function [36], and for small times this function is approximated as
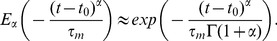
(14)


For the simulation of this model presented in our work we used the full Mittag-Leffler function instead of this approximation. In Eq. 13 the term with the Mittag-Leffler function (right side and right term) represents the memory trace. As in the classical integrate and fire model when the voltage reaches 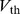 a spike is generated and V is reset to 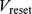 for a refractory period 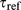. The subthreshold voltage is integrated using Eq. 13 with initial voltage 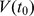 = 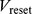 and new initial time 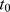. During each integration cycle the Mittag-Leffler function restarts from 0 since the initial time 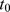 reset to a new value. We call this memory reset. Thus, this model wipes out the memory trace after every spike, in contrast to our model that integrates the entire voltage trace. As a result, the inter-spike intervals of the spike train of the analytic solution are equal. If the approximation (Eq. 14) is used to simulate the voltage, the firing rate can also be approximated analytically by combining Eq. 13 and Eq. 14 (see also [74]). Let 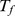 be the time when the voltage takes to increase from 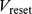 to 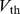 and to fire. The time 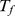 is given by [74]

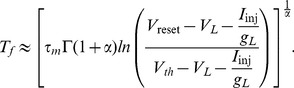
(15)Using the above the firing rate is approximated by
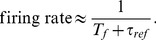
(16)


In the Results section we compare this analytical model with our model with memory reset (re-starting the memory trace after every spike) and with the full model (integrating the memory trace from the beginning of the simulation).
